# The NS4A Cofactor Dependent Enhancement of HCV NS3 Protease Activity Correlates with a 4D Geometrical Measure of the Catalytic Triad Region

**DOI:** 10.1371/journal.pone.0168002

**Published:** 2016-12-09

**Authors:** Hamzah A. Hamad, Jeremy Thurston, Thomas Teague, Edward Ackad, Mohammad S. Yousef

**Affiliations:** 1 Department of Physics, College of Arts and Sciences, Southern Illinois University Edwardsville, Illinois, United States of America; 2 Biophysics Department, Faculty of Science, Cairo University, Giza, Egypt; University of Nebraska-Lincoln, UNITED STATES

## Abstract

We are developing a 4D computational methodology, based on 3D structure modeling and molecular dynamics simulation, to analyze the active site of HCV NS3 proteases, in relation to their catalytic activity. In our previous work, the 4D analyses of the interactions between the catalytic triad residues (His57, Asp81, and Ser139) yielded divergent, gradual and genotype-dependent, 4D conformational instability measures, which strongly correlate with the known disparate catalytic activities among genotypes. Here, the correlation of our 4D geometrical measure is extended to intra-genotypic alterations in NS3 protease activity, due to sequence variations in the NS4A activating cofactor. The correlation between the 4D measure and the enzymatic activity is qualitatively evident, which further validates our methodology, leading to the development of an accurate quantitative metric to predict protease activity *in silico*. The results suggest plausible “communication” pathways for conformational propagation from the activation subunit (the NS4A cofactor binding site) to the catalytic subunit (the catalytic triad). The results also strongly suggest that the well-sampled (via convergence quantification) structural dynamics are more connected to the divergent catalytic activity observed in HCV NS3 proteases than to rigid structures. The method could also be applicable to predict patients’ responses to interferon therapy and better understand the innate interferon activation pathway.

## Introduction

The hepatitis C virus (HCV) is a significant worldwide health concern, afflicting up to 170 million people [1, 2]. It exists in developed countries in North America, Europe, and Japan, and appears most frequently in regions of Africa and the Eastern Mediterranean. Although 70–80% of cases are asymptomatic [3], some cases are associated with serious liver diseases such as cirrhosis or hepatocellular carcinoma [4]. Furthermore, HCV is a major cause of type I mixed cryoglobulinemia, which occurs in 10% of patients [5]. In total, there are seven different known genotypes of HCV, numbered 1 to 7. Different genotypes are found in different geographic areas. HCV-1 is a genotype primarily found in America, Europe, and Japan. HCV-1a is the predominant strain in North America and Northern Europe, while HCV-1b is the most common subtype in Japan and Eastern Europe [6]. HCV-3a and HCV-4a account for about 70% of HCV infections in Pakistan [7, 8] and 90% of HCV infections in Egypt, respectively [9–11]. Due to its prevalence in more developed areas, the HCV-1 genotype underwent more intensive research that has resulted in the development of effective antiviral drugs [12–14]. Genotypes found in developing countries have fewer and more expensive treatments due to the lack of research focused on these particular genotypes [15].

Once infected with HCV, host cells induce interferon (IFN)-mediated immune defense to limit virus replication. In response, HCV uses various strategies to evade the host’s innate immune system. One strategy involves HCV NS3/4A protease, which cleaves the Cardif and TRIF proteins to cripple the innate immune system’s type I IFN induction signaling and its capacity to recognize antigens. Therefore, the role that the hepatitis C virus (HCV) NS3/4A protease plays in crippling the signaling pathway concerned with the production of alpha/beta interferon (IFN-α/β) indicates a link between NS3/4A proteolytic activity and a patient's response to IFN-based therapy. Likewise, a correlation exists between the efficiency of NS3/4A protease Cardif cleavage and IFN therapy outcome [16]. In addition, and due to its critical role in protein replication, HCV NS3/4A protease is often targeted by antiviral drugs and other inhibitors [17–22]. These interactions with inhibitors have been intensively studied using computational tools [23–28].

The HCV NS3 protease, a viral serine protease with a chymotrypsin-like fold, which is activated by a bound NS4A (a peptide cofactor) [29] is distant from the catalytic site. The catalytic site of the protease includes three essential residues, histidine-57, aspartic acid-81, and serine-139 [30], commonly referred to as the catalytic triad and which performs general acid-base catalysis on target peptides [31]. Deletion analysis showed that the central region, a 13-residue segment (residues 21 to 34) of the NS4A, a 54-residue protein, is essential and sufficient for the cofactor activating function [32–37]. The NS4A forms a non-covalent complex with the N-terminal 22 residues of NS3 serine protease [32–34, 36–39]. Substitutions that disrupted the interaction between NS3 and NS4A result in reduction or loss of protease activity, suggesting that formation of an NS3-NS4A complex could be a pre-requisite for a functional serine protease [33, 38–44].

In our earlier work [44, 45], we developed a 4D computational methodology to examine the active site geometry in HCV NS3 protease from genotypes 1b, 3a and 4a. The results indicated a remarkable correlation between the experimentally measured pan-genotypic catalytic activities of HCV NS3 proteases and a 4D geometrical measure, obscured in the rigid structures, which emerged from our computational methodology. We concluded that the pan-genotypic variations in catalytic activities could be predicted and explained through the variant 4D dynamic behavior of the catalytic triads, notwithstanding the almost identical rigid structures of the proteases.

Here we investigate the sensitivity of our newly developed computational method [44, 45] in probing the effect of amino acid variations in the NS4A cofactor on the enzymatic activity of HCV NS3 protease genotype 3. We simulate the NS3 with NS4A cofactor combinations, whose catalytic activities were measured experimentally [46]. When an NS4A cofactor from a highly active genotype 1b protease was exchanged with a 3a cofactor from a weakly active 3a protease, a dramatic increase in the catalytic activity occurred in the hybrid 3a* protease, bound to the 1b cofactor [46]. Our computational methodology, based on 3D structural modeling and rigorously sampled molecular dynamic simulations is shown to be accurate enough to, at least, qualitatively predict this intra-genotypic, cofactor dependent, catalytic enhancement. In addition, motion correlation analysis between residues in the NS4A cofactors and residues V55-D81 in the protease corroborate NMR data [47] and suggest a plausible “communication” gateway, which could transmit structural fluctuations from the activation subunit (cofactor binding site) to the catalytic subunit (triad region). As mentioned before, the catalytic activity of NS3 protease is known to correlate with patients responses to interferon therapy [48], viral persistance [49] and in some cases with viral virulence [50]. Therefore, our method represents a step towards the development of a powerful predictive metric, which could inform treatment regimens and epidemiology studies. Moreover, the results are expected to have a broader impact on our understanding of catalysis and inhibition in the ubiquitous family of serine proteases.

## Methods

### 3D Structure Prediction and Validation

The 3D structure of HCV-3a and HCV-1b NS3 proteases with bound NS4 cofactors were predicted and validated as previously detailed [45], using the programs LOOPP [51], SWISS-MODEL [52–56], the CCP4 program suite 6 [57, 58] the GROMOS96 program, an implementation of the Swiss-pdb viewer [59], Procheck, What_Check, ERRAT, Verify_3D, Prove [3, 60–63], MDWeb [64].

### Molecular Dynamics Simulations

The molecular dynamics simulations (MD) were performed using NAMD 2.9 under the CHARMM27 force field for proteins [65–67] using the same minimization, heating and production methodology as detailed previously [45].

The system was then simulated for 90 ns. Only equilibrated time frames were used for the measurements: 10–90 ns. The equilibrium state of the protease was determined by the RMSD of the entire protein's backbone. Multiple copies of each protease, which included the cofactor and a zinc ion (non-bonded), were run with different initial conditions to ensure that the results were well converged. All data presented are averaged over eight distinct runs in order to ensure a representative sample of the parameter space the protease explores. Further, the total equilibrated trajectory for each strain (10–90 ns) had their convergence quantified [68] using grcarma [69] with step sizes of 4 (smaller step size resulted in matrices too impractical to use), 10 and 20 on the whole protein’s backbone including the cofactor (NS4A).

In order to quantify the relative positions of the three catalytic residues (H57, D81 and S139) simultaneously, three atoms were used as vertices of a triangle. The atoms chosen were O_δ2_ of D81, N_δ_1 of H57 and O_γ_ of S139. The area is calculated by $\text{A}=\frac{\left|\vec{R_{1}}\;*\;\vec{R_{2}}\right|}{2}$ where $\vec{R_{1}}$ is the vector from O_γ_ of S139 to N_δ1_ of H57 and $\vec{R_{2}}$ is the vector from O_γ_ of S139 to O_δ2_ of D81. The distribution of the area of the triangle was monitored during the course of the simulation.

The residue-residue and backbone-backbone cross-correlations were calculated using the Linear Mutual Information [70–72] algorithm implemented in WORDAM. The energy calculations were done using the NAMD Energy plugin in VMD. RMSD and RMSF calculations were performed using VMD’s measure function [73].

## Results and Discussion

**Fig 1:**
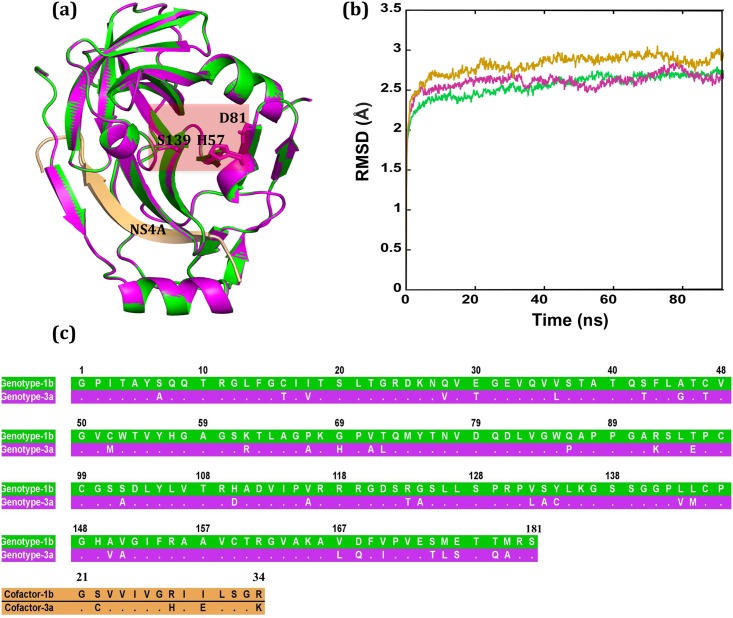
Comparison between the 3D structural models and dynamics of HCV-3a and the HCV-1b NS3 protease. (a) Structural models of HCV-1b (green) and HCV-3a (magenta) are superimposed. The transparent box highlights the catalytic triad (H57, D81, and S139). (b) Residue-average RMSD of Cα atoms for the models of HCV-1b (green), HCV-3a (magenta) and HCV3a* (gold, see methods) during the simulation. (c) The alignment of the amino acid sequences of HCV-1b (green), HCV-3a (magenta) NS3 proteases, as well as their corresponding NS4A cofactors. Dots show identical sequences.

The rigid backbone structures of HCV-1b and HCV-3a protease models are indistinguishable, with backbone RMSD around 0.2 Å (Fig 1A). The sequence identity between the two proteases is about 80% (Fig 1C). The conserved catalytic triad residues H57, D81, and S139 are positioned in a cleft between two β-barrels (Fig 1A) [47, 74, 75], forming a non-polar and shallow active site [31]. The rigid structures show that the active sites in both models are equally accessible. The structures also indicate that the main region of NS4A (residues 21–34) is buried within the protease to function as a fold-aiding cofactor (Fig 1A) [75]. None of the 181 amino acids exhibit steric clashes or stereochemical outliers, and Molecular dynamics (MD) simulations predict that both HCV-1b and HCV-3a proteases equilibrate at an average RMSD in the Cα positions of about 2.5 Å (Fig 1B).

**Fig 2:**
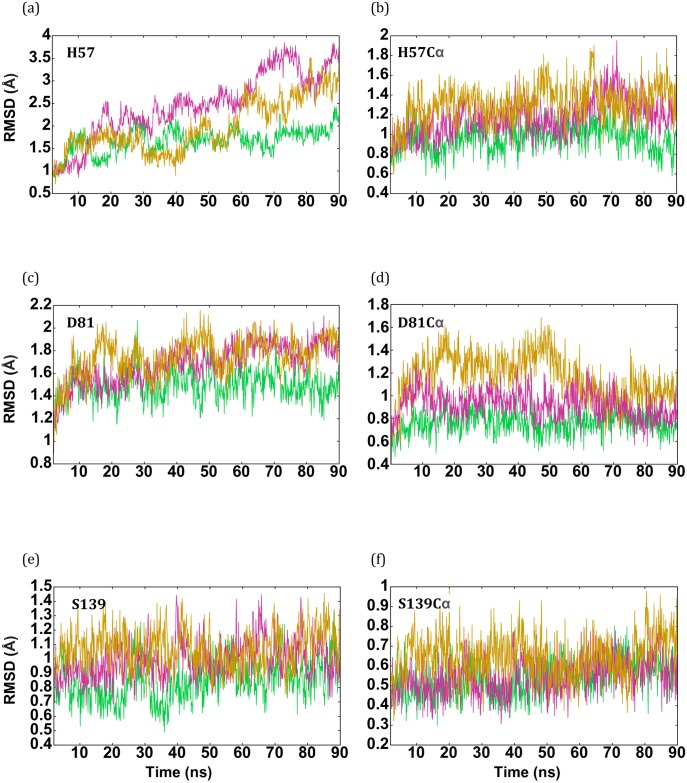
Comparison of the dynamical behavior of the catalytic triad residues among the protease models (HCV-1b, green, HCV-3a, magenta, and HCV-3a*, gold). RMSD values for each catalytic residue are shown for the entire residue (a, c, e) and the corresponding Cα atom (b,d,f).

**Fig 3:**
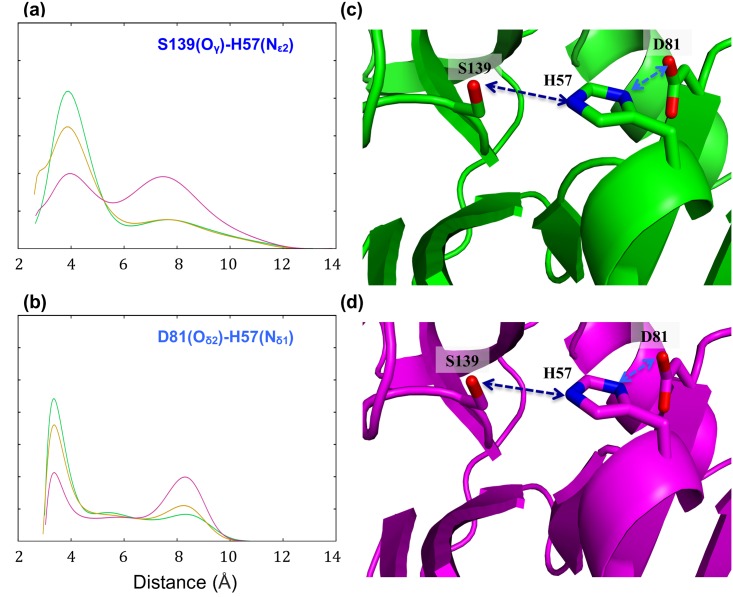
Dynamical behavior within the catalytic triad region of the protease models (HCV-1b, green, HCV-3a, magenta, and HCV-3a*, gold). The distance distribution profiles (a) between O_γ_ of residue S139 and N_ε2_ of residue H57, and (b) between O_δ2_ of residues D81 and N_δ1_ of H57, during the stimulation for the threading protease models (HCV-1b, green, HCV-3a, magenta and HCV-3a*, gold). Blue and cyan arrows indicate the selected distances in the rigid structures.

**Fig 4:**
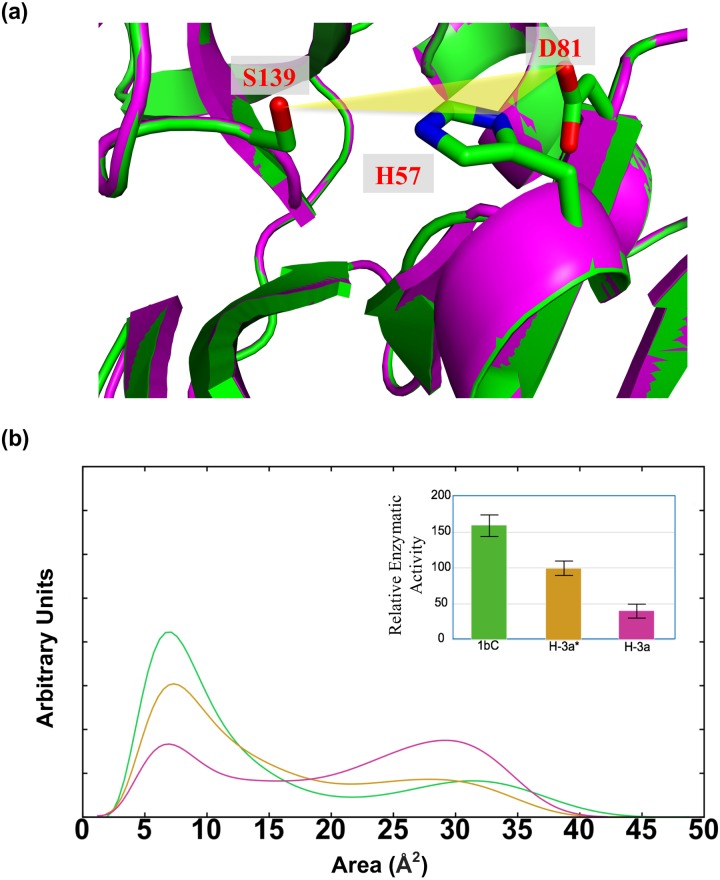
The conjoint dynamical behavior of the catalytic triad site expressed as the area of a triangle (yellow) whose vertices lie on each catalytic residue (a). (b) The area distribution profile of the triangle bridging the catalytic residues in the models (HCV-1b, green, HCV-3a, magenta and HCV-3a*, gold). The inset depicts the relative enzymatic activity of each protease variant, experimentally measured in Ref. [46]. The trend in enzymatic activities follows, at least qualitatively, the corresponding values of the area distribution profiles at around 7Å^2^.

However, MD simulations locally exhibit a genotype-dependent, divergent dynamics profile within the catalytic triad region, with HCV-1b protease being the most stable and the HCV-3a the most deviating (Figs 2, 3 and 4). These dynamic distinctions have a strong correlation with the alterations in catalytic activities (Fig 4B) and drug responsiveness to linear inhibitors observed in these two genotypes [19, 46]. In this regard, this result implies that the triad region’s intrinsic dynamics could directly predict HCV pan-genotype enzymatic activities and its subsequent physiological/clinical ramifications, such as the ability of host cells to elicit an innate immune response and respond to interferon based therapy [46, 48].

Our MD simulations also show that swapping HCV-3a NS4A cofactor for its HCV-1b counterpart in the HCV-3a variant, which we will refer to hereafter as HCV-3a*, restored most of the local stability in the catalytic triad region to a level comparable to that of 1b protease (Figs 2, 3 and 4). This restored local stability in HCV-3a* is shielded from the increased backbone motion (Fig 1B). This trend directly correlates with the experimental measurements of the catalytic activities [46] observed for these variants (Fig 4B). Using 4D simulation of the interactions between the catalytic residues following the same methodology we reported previously [44, 45], we examined the confined positional dynamics of the catalytic triad residues. In addition, we used the distance distribution profiles of catalytically significant distances as gauges of the 4D differences. The alpha carbons (Cα) of the catalytic residues S139 exhibit somewhat similar dynamics throughout the simulations for the variants (genotypes 1b and 3a and 3a*), while the Cα of the catalytic residues D81 and H57 in variants 3a and 3a* show a slight to moderate increase in RMSD of ~0.2 to 0.4 Å respectively, relative to genotype 1b (Fig 2A, 2B, 2C and 2D). Similarly, the RMSF of Cα atoms show no significant difference between the strains, demonstrating that the global motion is not indicative of the local dynamics at the catalytic triad region (S1 Fig).

As for the entire residues, H57 in both HCV-3a and HCV-3a* models demonstrate dynamic behaviors that differ from that of HCV-1b (Fig 2A). By averaging the results of eight distinct runs, the RMSD of H57 in the HCV-3a model was seen to deviate from that predicted through the HCV-1b template by up to 2 Å at various points during the simulation. A similar but less drastic trend is observed in genotype 3a* where the dynamics behavior is almost identical to that of 1b for two thirds of the simulation and deviates only at the last third. The distance distribution profiles between N_ε2_ of H57 and O_γ_ of S139, as well as between N_δ1_ of H57 and O_δ2_ of D81, for HCV-1b, HCV-3a protease models significantly vary in both peak value and width. In the HCV-1b model, the distance between O_δ2_ of D81 and N_δ1_ of H57 (green in Fig 3B) presents a sharp distribution with a peak value around 3.4 Å. In the model HCV-3a (magenta in Fig 3B), the corresponding distance distribution is bimodal, much broader, and distributed around 3.5 and 8.3 Å. In the model HCV-3a* (gold in Fig 3B), it is clear that the 1b trend was almost restored, evident in the recovery of the characteristic sharp peak around 3.4 Å, overlapping with the HCV-1b peak. Similarly, the distance between O_γ_ of S139 and N_ε2_ of H57 in HCV-1b (green in Fig 3A) exhibits a sharp distribution with a peak value around 4 Å. In the model HCV-3a (magenta in Fig 3A), the corresponding distance distribution is bimodal, much broader, and distributed around 4 and 7.5 Å (identical to what we observed previously [45]). In the model HCV-3a* (gold in Fig 3A), the 1b trend is almost restored. In order to help assess the relative positions of the three catalytic residues simultaneously, the combined dynamic behavior of the three catalytic residues as vertices of a triangle was also examined (Fig 4).

**Fig 5:**
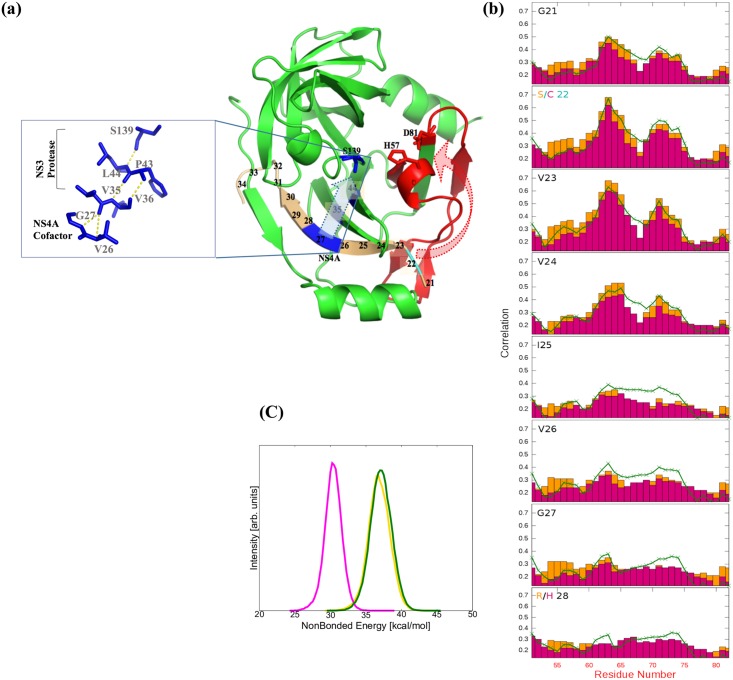
Putative communication pathways from the activation subunit (cofactor binding) to the catalytic subunit in NS3 protease. (a) An overall representative structure of HCV NS3 protease (green), the cofactor is shown in beige. The catalytic subunit (catalytic triad) is highlighted by a dotted ellipse. Inset: hydrogen bonding network between residues 27 and 26 in the NS4A cofactor and anti-parallel beta sheets in the NS3 protease, leading to residue S139 in the catalytic subunit. The red dotted arrow in (a) indicates a putative conformational pathway from site (C22_3a_/S22_1b_, cyan), in the cofactor to the catalytic triad through residues 51–81 (red). The blue dotted arrow in (a) indicates another putative pathway in blue from the NS4A cofactor leading to the catalytic residue S139 through the hydrogen bonding network shown in the inset. (b) Analysis of backbone correlated motions between residues 21–28 in the NS4A cofactor and residues 51–81 [red in (a)] in the NS3 protease. The backbone motion correlations are shown for NS3 proteases for the variants HCV-1b (green), HCV-3a (magenta) and HCV-3a* (gold). The correlation scale is set from 0.0 (for no correlation) to 1.0 (completely correlated). Swapping the 1b NS4A cofactor for the 3a cofactor resorted HCV-1b-like correlation in the variant HCV-3a*, particularly at site (C22_3a_/S22_1b_). Correlation is negligible for other cofactor mutations sites (K34_3a_/R34_1b_), (E30_3a_/I30_1b_) and (H28_3a_/R28_1b_); thus not shown. (c) The non-bonded energy distribution during the course of the simulation, for the cofactor residues S22_1b_, S22_3a_*and C_22_, following the same color code used in Fig 1b.

As previously described [45], we chose atoms O_δ2_ of D81, N_δ1_ of H57 and O_γ_ of S139 as vertices. During the course of the simulation, the distribution profiles of the area of the triangle show a single sharp peak in HCV-1b and a wide bimodal distribution in HCV-3a. In HCV-3a*, once again the recovery of the 1b trend is evident (Fig 4B). The area of the triangle characterizes a “catalytic plane” whose distribution profile could be predictive of alterations in the optimal catalytic geometries. In this sense, HCV-1b protease is predicted to be the most stable (most active) and HCV-3a is the least stable (least active) while HCV-3a* represents an intermediate state closer to HCV-1b. This is consistent with the observation that the catalytic activity of HCV-3a NS3 protease is significantly less than that of HCV-1b, while the catalytic activity of HCV-3a* is somewhat less than that of HCV-1b, but not as low as that of HCV-3a protease [46]. It is noteworthy to mention that the predicted difference in dynamics behavior for HCV-1b, HCV-3a and HCV-3a* (Figs 3, 4 and 5) is completely opaque in the rigid structures by the apparent similarity of the catalytic site. These results highlight the importance of utilizing molecular dynamics, among other methods, to investigate protease activity. In addition, the strong correlation between the predicted conformational stability of the catalytic triad region with the experimental values catalytic activity seen among HCV NS3 protease variants, paves the path for future predictive applications. Of a particular importance, the correlation between the catalytic efficiency of NS3/4A protease and patients’ response to IFN therapy [16]. In this regard, our methodology could provide a basis for accurate predictions of IFN treatment outcomes. It is evident that the NS3/4A protease interferes with and attenuates the signaling pathway involved in the production of IFN-α/β through cleaving essential intermediates. This suggests a relationship between NS3/4A protease activity and patients’ response to IFN based therapy [76].

### Putative Information Gateway(s) Between the Activation Subunit and the Catalytic Subunit

In an attempt to dissect the structure/function role of the NS4A cofactor in relation to the catalytic activity, we performed backbone motion cross-correlation analysis between individual residues in the cofactor bound to the structural models (1b, 3a and 3a*) and a contiguous segment in the protease, encompassing residues V51-D81. This segment includes two catalytically essential amino acids, namely H57 and D81 (Fig 5). Nuclear Magnetic Resonance (NMR) studies showed that NS4A binding somehow induces the proper alignment of the catalytic triad as demonstrated by key Nuclear Overhauser Effects (NOEs) and the downfield chemical shift of histidine-57 backbone amide proton [47]. A comparison of chemical-shift differences between NS3 protease and the NS3 protease-NS4A complex showed extensive chemical-shift changes for residues V51-D81, indicating a propagation of non-local structural changes induced upon NS4A cofactor binding and propagated beyond the cofactor-protease binding site. This is supported by crystallographic data, which revealed extensive structural rearrangements of the strand and loop regions that are formed. In addition, this segment is known for being the “first respondent” to NS4A cofactor binding as shown by NMR [47]. Correlated motions in proteins are known to transmit structural information, making the analysis of which essential to clarify pathways that link distant regions in the protein [77]. Our backbone motion correlation results (Fig 5B) show that swapping the HCV-3a NS4A cofactor with HCV-1b NS4A cofactor in the weakly active HCV-3a protease, enhanced the motion cross-correlation between the cofactor residues and the “respondent” segment V51-D81 to a level comparable to that predicted for the highly active HCV-1b protease. Remarkably at the mutation site (C22 in 3a/S22 in 1b) in the cofactor, which is at a close proximity to the “responding” segment (Cyan in Fig 5A), the level of motion correlation is almost identical to that of 1b protease. Therefore, the 1b cofactor restored motion correlations that were absent in the weakly active 3a protease. Although serine and cysteine residues differ only in the replacement of the O-H group in the former and of the S-H group in the latter, they exhibit rather different VROA spectra [78]. We hypothesize that a C/S mutation in the cofactor, replacing the heavy sulfur with the lighter oxygen on the side chain, affects the vibrational frequency and the backbone local flexibility around the residue. This altered flexibility may trigger an activation pathway through residues V51-D81, which ultimately align/misalign the catalytic triad. Preliminary evidence supporting this hypothesis is found in the non-bonded energy of the cofactor’s C/S residue with the rest of the protein (shown in Fig 5C). While the actual values for the energy are not completely relevant, the almost 20% downshift in the peak value substituting Ser with Cys, is suggestive of a mechanism by which the loosely bound Cys weakens the communication with the “respondent” V51-D81 region. This ultimately could misalign the catalytic residues D81 and H57. This is all rather speculative at this stage and final confirmation awaits more intensive investigations. In addition, it remains to be seen whether this enhanced correlation is mainly due to the C/S mutation, or if the effect is cumulative/additive due to other variations in the cofactor sequence. Another possible “communication gateway” is through the hydrogen-bonding network incorporating the NS4A cofactor into the protease antiparallel beta sheets (Fig 5A). Even though our results indicate no significant preferential changes in bonding/dynamics though this pathway for the genotypes at hand (i.e. genotypes 1b, 3a and 3a*) upon swapping cofactors, this does not rule out the possibility that this gateway is permeable to structural fluctuations in other sub-strains/cofactor combinations.

### Assessing Convergence and Conformational Sampling

**Fig 6:**
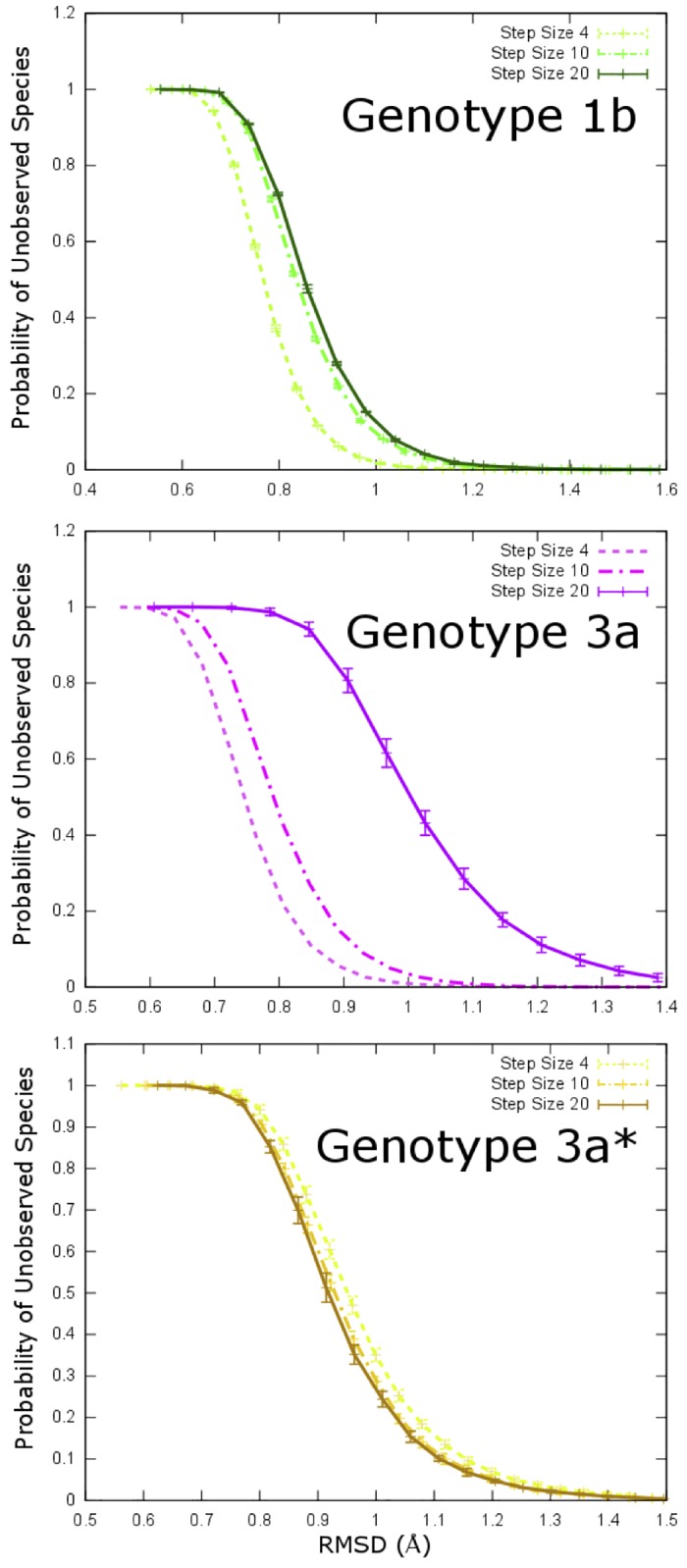
Convergence and sufficient sampling. Results from the application of Good-Turing statistics to estimate the extent of conformational sampling in HCV NS3 protease models for genotypes (1b:green, 3a:magenta 3a*:gold). The probability of an unobserved structure as a function of the backbone RMSD difference from the closest already observed structure. The solid line is using a stride size through the trajectory of 20, the dotted line is using a stride size of 10 and the faint line is using a stride size of 4 (the largest which could be reasonably handled). (The lack of error bars in 6b is due to the sub-sampling factor being exactly 1 [68].

As our progress towards augmenting a robust predictive metric for serine protease enzymatic activity, we save no effort to ensure that the conformational sampling scheme is rigorous. We do not rely exclusively on the equilibration trends seen in Fig 1B for the assessment of structural convergence and adequacy of the sampling. We also measure the probabilities of unobserved species as a function of backbone RMSD from all observed conformations [68]. For genotype 1b, the probability of unobserved species with 0.7 Å RMSD from all observed conformation is 0.8 and next to zero for an RMSD of 1.2 Å or higher (Fig 6A). In other words, for genotype 1b, one would not expect to see deviations of greater than 1.2 Å even with a longer simulation. The HCV-3a* protease exhibits a small but noticeable decrease in the probability of observing new conformations with modest backbone deviations (~1 Å) as the step size decreases and thus it is also well sampled (Fig 6C). Evidently, the HCV-3a* protease is significantly better sampled than that of HCV-1b as even the coarser step size of 20 is sufficient to “screen” the backbone’s conformational population. This suggests that the HCV-3a* protease is indeed well sampled within the simulated trajectories. The HCV-3a protease exhibits a large decrease in probability when the step size is lowered from 20 to 10, but only a small change from a step size of 10 to 4 (Fig 6B). Thus, the HCV-3a protease’s flexibility, despite having comparable overall backbone RMSD to that of HCV-1b and HCV-3a* (Fig 1B), has it exploring wider conformational space compared to other strains. The close agreement between step sizes 4 and 10 suggests that overall, once all data are included, a good sampling of the protease’s conformations has been achieved. The step sizes indicate the number of frames in each input trajectory skipped during the probability calculations. In general, a larger step size, or equivalently, a coarser analysis results in an overestimate of the probability at each value of RMSD. Additionally, the error bars of the step size 20 curve are much larger than those found in Fig 6A and 6C. This fact points to a strong dependence of the resulting probability curves on the starting points in the sampling process. More specifically, the results of the statistical analysis for a step size of 4 will differ noticeably depending on whether the first, fifth, ninth, etc. frames are used instead of the second, sixth, tenth, and so forth. From this, it can be concluded from the small error bars in Fig 6A and 6C that the HCV-1b and HCV-3a* strains are insensitive to the initial choice of frame. All of these behaviors are likely to be a reflection of the higher degree of stability (evident from the ease of sampling) in the HCV-3a* protease relative to its HCV-3a and even HCV-1b counterparts (it should be noted that the lack of error bars on the smaller step sizes for the HCV-3a are due to the subsampling factor being equal to 1.

In summary, we have demonstrated that the enzymatic activity of HCV NS3 protease could be predicted accurately, at least qualitatively, at both pan- and intra- genotypic levels. Our 4D predictive methodology ensures rigorous conformational sampling and correlates well with experimental data. The results presented here represent a significant step forward towards the development of fast and accurate computational metric(s) for predicting enzymatic activity *in silico*.

## Supporting Information

S1 Fig RMSF of the C_α_ atom for the NS3 proteases, HCV-1b (green), HCV-3a (magenta), HCV-3a* (gold) residues. The inset shows RMSF for the corresponding NS4A cofactors residues as numbered in Fig 1c.
(TIF)
