# Supplementary figures and images for: The NS4A Cofactor Dependent Enhancement of HCV NS3 Protease Activity Correlates with a 4D Geometrical Measure of the Catalytic Triad Region
Source: PLoS One. 2016 Dec 9;11(12):e0168002. doi: 10.1371/journal.pone.0168002 (PMC5148068; doi:10.1371/journal.pone.0168002)

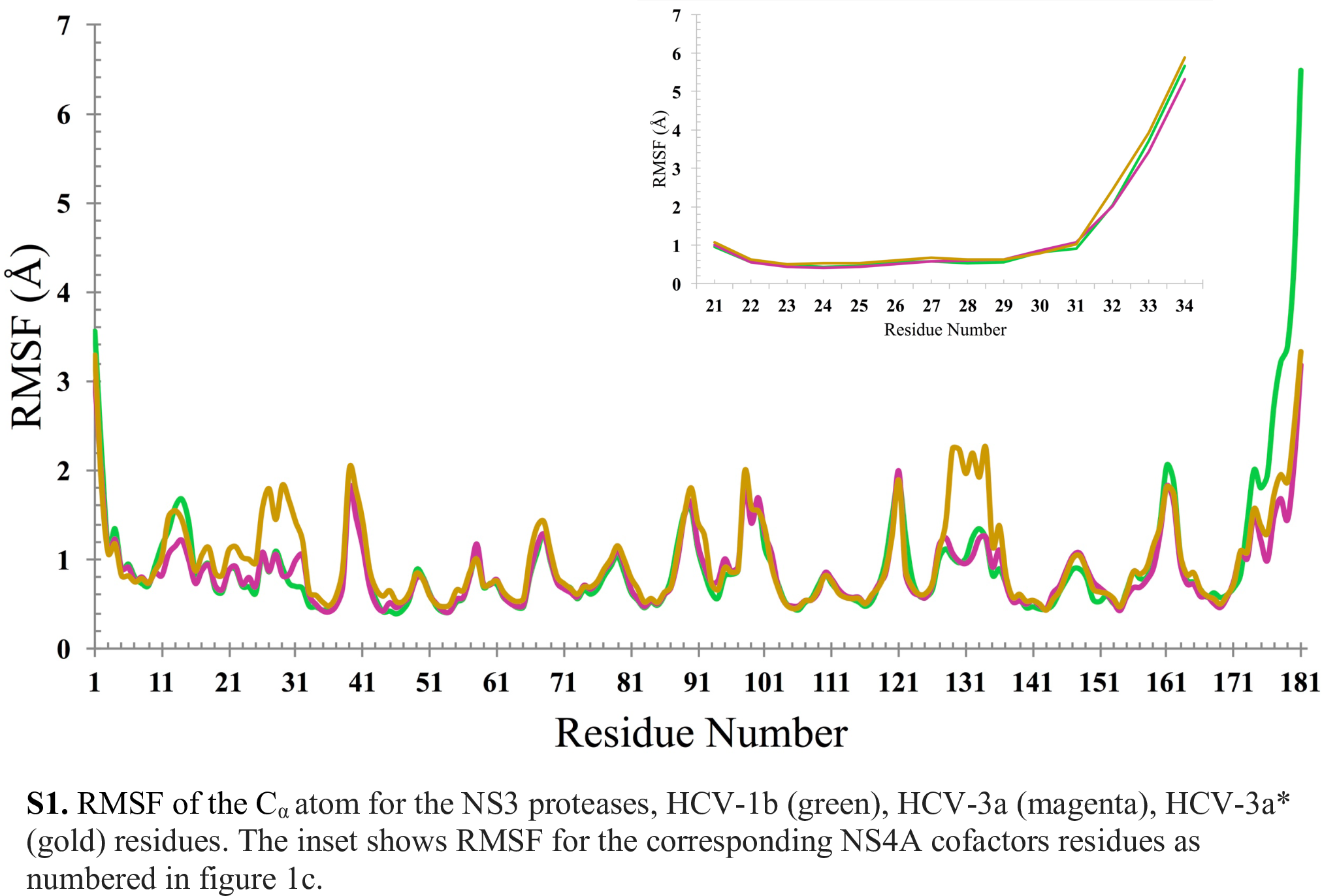

Supplement: S1 Fig — The inset shows RMSF for the corresponding NS4A cofactors residues as numbered in Fig 1c. (TIF) [file pone.0168002.s001.tif]
